# Application of network pharmacology in traditional Chinese medicine for the treatment of digestive system diseases

**DOI:** 10.3389/fphar.2024.1412997

**Published:** 2024-07-17

**Authors:** Shihao Zheng, Yijun Liang, Tianyu Xue, Wei Wang, Size Li, Peng Zhang, Xiaoke Li, Xu Cao, Qiyao Liu, Wenying Qi, Yongan Ye, Xiaobin Zao

**Affiliations:** ^1^ Dongzhimen Hospital, Beijing University of Chinese Medicine, Beijing, China; ^2^ Liver Diseases Academy of Traditional Chinese Medicine, Beijing University of Chinese Medicine, Beijing, China; ^3^ Xiyuan Hospital of China Academy of Chinese Medical Sciences, Beijing, China; ^4^ First Affiliated Hospital of Hebei University of Chinese Medicine, Shijiazhuang, China; ^5^ Dongfang Hospital, Beijing University of Chinese Medicine, Beijing, China; ^6^ Key Laboratory of Chinese Internal Medicine of Ministry of Education and Beijing, Dongzhimen Hospital, Beijing University of Chinese Medicine, Beijing, China

**Keywords:** network pharmacology, digestive system diseases, traditional Chinese medicine, compounds, targets

## Abstract

With the general improvement in living standards in recent years, people’s living habits, including their dietary habits, have changed. More people around the world do not follow a healthy diet, leading to an increase in morbidity and even mortality due to digestive system diseases, which shows an increasing trend every year. The advantage of traditional Chinese medicine (TCM) in treating digestive system diseases is evident. Consequently, the mechanisms of action of single Chinese herbs and compound Chinese medicines have become the focus of research. The research method of the network pharmacology system was highly consistent with the holistic concept of TCM, and provided a new perspective and theoretical basis for basic research on digestive system diseases. This article summarizes the common databases currently used in research on TCM. It also briefly introduces the basic methods and technologies of network pharmacology studies. It also summarizes the advancements of network pharmacology technology through a comprehensive literature search on PubMed. Based on this analysis, we further explored the role of TCM in treating digestive system diseases, including chronic gastritis, gastric cancer, ulcerative colitis, and liver cirrhosis. This study provides new ideas and references for treating digestive system diseases with TCM in the future and serves as a reference for relevant researchers.

## Introduction

Network pharmacology as an emerging discipline was first proposed by Hopkins, a British pharmacologist, in Nature Biotechnology in 2007 (Hopkins, 2007). Under the premise of the rapid development of network databases, network pharmacology is based on the theories of pharmacology, bioinformatics, and other disciplines. Further use of visualization technology, high-throughput technology, network analysis, and other methods could explore the effective mechanisms of single medicines or compounds of TCM in treating diseases. From a macro perspective, these methods elucidated the interaction mechanisms between single herbs or compounds in TCM and diseases. Furthermore, this comprehensive approach highlighted the characteristics of multiple compounds, targets, and pathways (Hopkins, 2007; Li and Zhang, 2013; Wu, et al., 2016; Zhang, et al., 2019a).

Network pharmacology is connected with systemic biomedical technology. Through computer software and the TCM database websites, the unique advantages of TCM in treating diseases have been combined with single medicines or compound medicines used in TCM. Until now, numerous active compounds or compound medicines used in TCM have been analyzed by the network pharmacology method, and the key action mechanisms for treating diseases have been discussed. These studies of network pharmacology provided a better reference for treating diseases and for the production of new clinical medicines. Thus, the theory of “medicine–compound–target–disease” in network pharmacology coincided with the holistic concept proposed by TCM (Hopkins, 2008). Nowadays, the application scope of network pharmacology is increasing, which effectively explores the pharmacological role and mechanism of TCM in the treatment of diseases, studies the application scope of TCM in the treatment of diseases, and effectively analyzes the theory of TCM (Liu, et al., 2017; Zhan and Su, 2018; Guo, et al., 2019; Han, et al., 2019). This paper used network pharmacology to review the research results and *status quo* of TCM in the treatment of digestive system diseases, to identify problems in this research field, and to provide new ideas and methods for the prevention and treatment of digestive system diseases in the future.

**FIGURE 1:**
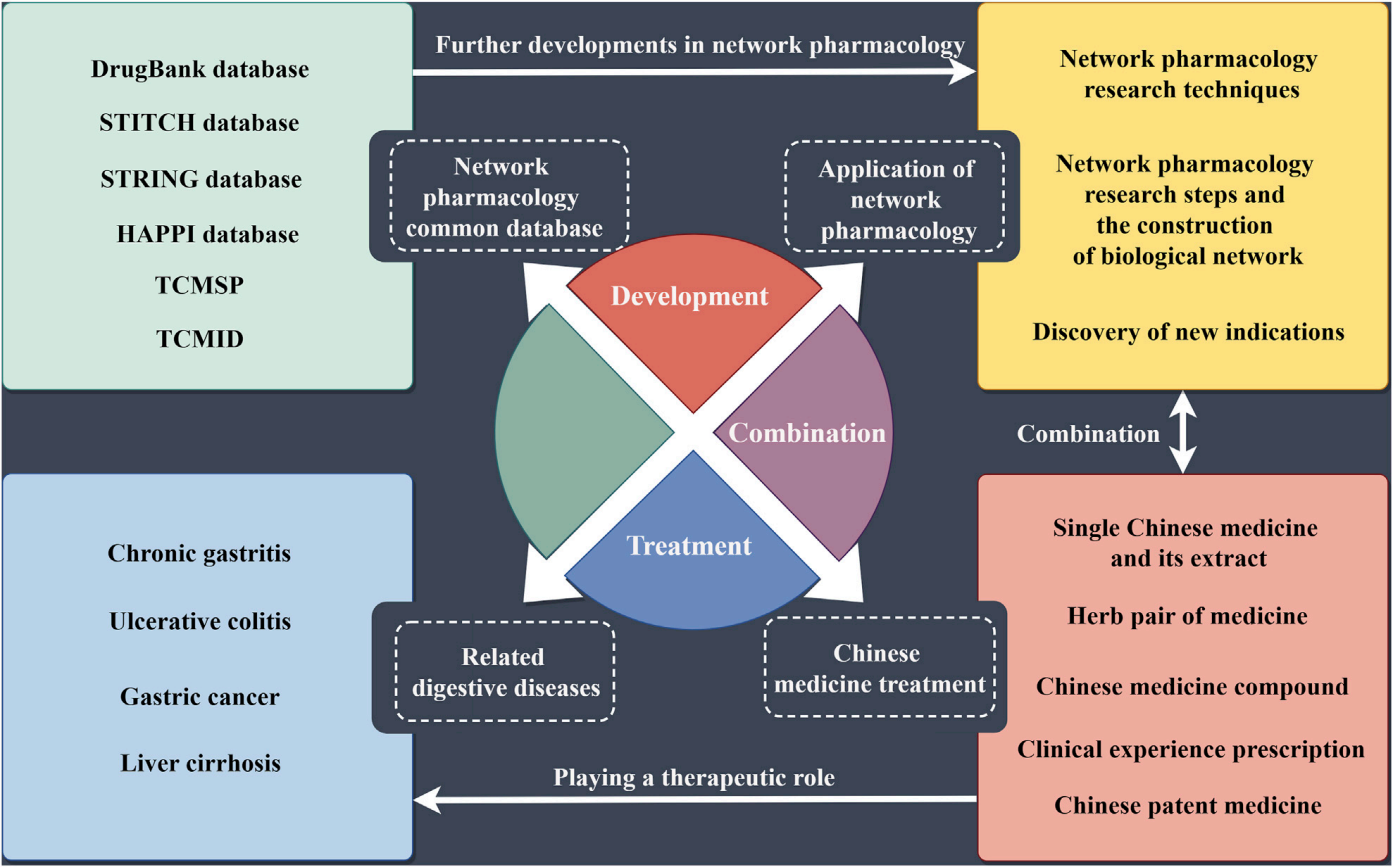
Relationship between digestive diseases and network pharmacology.

Digestive system diseases belong to the field of internal medicine and include conditions affecting the digestive tract, digestive glands, mesentery, and peritoneal organs. The digestive tract is composed of the mouth, esophagus, throat, stomach, small intestine, large intestine, and anus (Zhu, et al., 2017). Common diseases in this category mainly include chronic gastritis, gastric cancer, liver cirrhosis, and ulcerative colitis. China is recognized as a high-risk country for esophageal cancer (Liu, et al., 2022). In China, malignant tumors of the digestive system are the most prevalent type of cancer, whereas in Western countries, inflammatory bowel diseases (IBDs) are more common (Park and Cheon, 2021). The burden of digestive system diseases is significant, with high morbidity and mortality rates. For instance, liver cancer and stomach cancer are among the leading causes of cancer-related deaths worldwide. Chronic conditions such as gastroesophageal reflux disease (GERD) and ulcerative colitis severely impact the quality of life and require long-term management. Current treatment methods for digestive system diseases include medication, lifestyle changes, and surgical interventions. However, these treatments have limitations. For example, medications for GERD often need to be taken long term and could have side effects. Surgical treatments for cancers of the digestive system, while potentially curative, come with risks and potential complications. Furthermore, treatments for chronic conditions such as IBD often involve immunosuppressive drugs that have significant adverse effects and may not be effective for all patients (Feng, et al., 2022). The symptoms of digestive system diseases are diverse and can often be accompanied by symptoms of other systemic diseases. Therefore, it is crucial to fully understand the characteristics of these symptoms, the accompanying symptoms, and the appropriate medication for accurate diagnosis and treatment. Studies found that sleep quality could also affect the pathogenesis and symptoms of digestive system diseases (Orr, et al., 2020). This highlighted the close relationship between digestive system diseases and other conditions, emphasizing the importance of these diseases for public health (Figure 1). In conclusion, digestive system diseases pose a significant health burden due to their high prevalence, complex symptoms, and the limitations of current treatment options. A comprehensive understanding of these diseases and ongoing research into more effective treatments are essential to improving patient outcomes and quality of life.

### Common network pharmacology databases

#### DrugBank database

The DrugBank (https://go.drugbank.com/) database (Wishart, et al., 2006) is a comprehensive, freely accessible database that serves as a unique chemoinformatics and bioinformatics tool for retrieving information about drugs and drug targets. It combines comprehensive drug target information with detailed drug data, functioning like a drug encyclopedia. The majority of the drugs listed in Wikipedia are included in the DrugBank database, which has facilitated the discovery and repurposing of existing drugs to treat new or rare diseases more quickly. In network pharmacology, the DrugBank database is primarily used to identify disease target genes. The researchers then took the intersection of these target genes with the genes of the TCM compounds for further analysis. This process helps understand the molecular mechanisms of TCM treatments and validate their efficacy through scientific data. For example, a recent study used network pharmacology to analyze chronic atrophic gastritis, utilizing the DrugBank database to obtain target genes associated with the condition. Subsequent analysis and validation explored the mechanisms of action of TCM molecules on the disease and their interactions with disease targets (Weng, et al., 2023). Another study combined network pharmacology and experimental validation to investigate the molecular mechanisms of the anti-colorectal cancer activity of *Curcuma aromatica* Salisb. In this research, scholars used the DrugBank database to search for FDA-approved anti-colorectal cancer drugs and their targets for subsequent analysis (Yin, et al., 2024).

#### STITCH database

The STITCH (search tool for interactions of chemicals, http://stitch.embl.de/) database (Kuhn, et al., 2008) contains known and predicted chemical compounds and protein interactions, including functional and physical interactions. The STITCH database is very informative, consisting of information on 2.6 million proteins and 390,000 small-molecule interactions. The researchers can determine chemical compounds with similar molecular structures by inputting the chemical molecular structures of the ingredients. The targets of these structurally similar chemical compounds could be considered putative targets of the chemical ingredient to be determined. The data sources of the STITCH database include knowledge transfer between species, computer prediction, text mining, and integration of other databases. The biggest advantage of this database is its extensive data and structural similarity. Regarding the application of the STITCH database in network pharmacology, researchers primarily use it to screen for the corresponding targets of active ingredients in TCM. The STITCH database includes both known interactions and predicted targets of active compounds in TCM based on the chemical similarity and pharmacophore model (Cao, et al., 2021; Chen, et al., 2023). For example, in a study comparing the molecular mechanisms of Fuzi Lizhong pills and Huangqin decoction in treating cold and heat syndromes of ulcerative colitis, researchers used the STITCH database to identify targets of active compounds for subsequent analysis (Hu, et al., 2023). Additionally, in a study on the compounds and mechanisms of *Solanum nigrum* in the treatment of colorectal cancer, the authors used the STITCH database to screen target genes of active compounds based on chemical similarity and pharmacophore models, providing a foundation for further steps (Chen, et al., 2023).

#### STRING database

The STRING (https://string-db.org/) database (Snel, et al., 2000) is a source of known or predicted interactions between proteins, providing a comprehensive overview of the many interactions and functional synergies occurring between proteins as well as a background for systems biology. The STRING database aims to provide an integrated and critical assessment of protein–protein interactions (PPIs), including functional and physical associations. STRING offers a broader perspective on multispecies interactions, with a wide range of network visualizations and functional analysis tools. In network pharmacology, the STRING database played a crucial role in constructing PPI networks from TCM active molecules to disease targets. By utilizing the PPI networks generated from the STRING database, researchers were able to identify and filter key targets for subsequent analyses. This process aided in understanding the mechanisms of action of TCM and identifying potential therapeutic targets for various diseases. One study used network pharmacology and molecular docking to investigate the mechanism by which curcumin inhibits esophageal squamous cell carcinoma. After constructing a PPI network using the STRING database, the researchers identified the key targets of curcumin in inhibiting esophageal squamous cell carcinoma. Subsequent validation work was based on these identified targets (Wang, et al., 2024). Additionally, a network pharmacology study on the anti-gastric cancer effects of *Bidentis bipinnata Herba* also employed the STRING database to construct relevant PPI networks. Hub genes were identified through this process, followed by subsequent validation work based on these findings (Guo, et al., 2024).

#### HAPPI database

The HAPPI (http://discovery.informatics.uab.edu/HAPPI) database (Chen, et al., 2009) is the most comprehensive database for human PPI data to date, containing 2,922,202 unique PPIs. The HAPPI database collects 23,060 human proteins, including functional and physical interactions. It is favored by many scholars in the field of bioinformatics and provides important support for the study of network pharmacology in TCM. While the use of the HAPPI database is not as widespread as that of STRING, it focuses specifically on human protein interactions, ensuring detailed and rigorous data quality. In network pharmacology studies, the HAPPI database plays a crucial role in constructing relevant PPI networks. For instance, in a study investigating the treatment of colitis with dragon’s blood tablets, researchers used the HAPPI database to collect PPI network data, which provided a foundational basis for subsequent studies on the medicine’s mechanism of action in treating the disease (Xu, et al., 2014).

#### TCMSP database

TCMSP (traditional Chinese medicine systems pharmacology database and analysis platform, http://tcmspw.com/tcmsp.php) (Ru, et al., 2014) uses the HIT database prediction algorithm, through which the specific relationship between medicine targets can be obtained, and the disease information in the database comes from the PharmGKB database and TTD database. The key compounds and target information of TCM can be retrieved and screened in the TCMSP database to match the target of the disease. The database contains 499 kinds of TCM drugs, 837 related diseases, 29,384 kinds of compounds, and 3,311 targets. It is a common tool for screening ingredients and targets of TCM in network pharmacology. TCMSP focuses more on the pharmacological properties of TCM ingredients and systems pharmacology research, making it suitable for analyzing the multitarget mechanisms of these ingredients. TCMSP is widely used in network pharmacology research (Wang, et al., 2022a; Cao, et al., 2022). Its primary role is to screen the active ingredients of TCM and subsequently identify the corresponding targets of these active ingredients. For example, in the study by Hu et al., in which network pharmacology was used to explore the treatment of non-alcoholic fatty liver disease with Xiaochaihu decoction, the TCMSP database was used to collect data on active compounds and related targets of seven TCM drugs. Hu and his team then conducted further analysis based on these data (Hu, et al., 2020). Another network pharmacology analysis related to the treatment of ulcerative colitis using *Fructus mume* and *Rhizoma coptidis* similarly utilized TCMSP to screen the active ingredients of these TCM drugs and identify the corresponding targets. These data served as the basis for subsequent research steps (Yang, et al., 2023).

#### TCMID database

TCMID (traditional Chinese medicine integrated database, http://www.megabionet.org/tcmid/) (Xue, et al., 2013) is the world’s largest computerized TCM database for medicine screening, containing 3,791 diseases, 47,000 prescriptions, 8,159 TCM drugs, 6,828 medicines, 25,210 compounds, and 17,521 target genes. It provides a Java-based, web-based analysis tool. The TCMID database records important TCM-related information collected from various sources. Users can use text mining to correlate the information in TCMID with the OMIM, DrugBank, and PubChem databases. TCMID is frequently used in network pharmacology analysis and is recognized by many scholars. The TCMID database serves a similar role as the TCMSP database, primarily screening for active ingredients of TCM drugs and identifying the corresponding targets of these active compounds. For instance, in a study investigating the appetite regulation mechanism of the Chinese medicine jujube using network pharmacology, the authors used both the TCMSP and TCMID databases to obtain data on active compounds and conduct further analysis (Zhu, et al., 2022). Additionally, in a network pharmacology study on the treatment of constipation with TCM, the authors utilized both the TCMSP and TCMID databases to acquire data on active compounds and targets, forming the basis for subsequent analysis (Xu, et al., 2022) (Table 1).

**TABLE 1:** Public databases related to TCM network pharmacology.

Type		Name	Description	Website of database or tool	References
Databases	Drug related databases	DrugBank	A database for retrieving information on drugs and drug targets, known as the drug encyclopedia.	https://go.drugbank.com/	Wishart et al. (2006)
		STITCH	A database of known and predicted chemical compounds and protein interactions.	http://stitch.embl.de/	Kuhn et al. (2008)
	Target related databases	STRING	A database of known or predicted interactions between proteins.	https://string-db.org/	Snel et al. (2000)
		HAPPI	The most comprehensive human PPI database to date.	http://discovery.informatics.uab.edu/HAPPI	Chen et al. (2009)
	TCM related databases	TCMSP	A database that can retrieve and screen the key compounds and target information of TCM.	http://tcmspw.com/tcmsp.php	Ru et al. (2014)
		TCMID	A computerized TCM database for medicine screening.	http://www.megabionet.org/tcmid/	Xue et al. (2013)

### Application of network pharmacology in traditional Chinese medicine research

#### Network pharmacology research techniques

Supported by emerging technologies such as molecular interaction technology, high-throughput technology, and omics technology, network pharmacology further improved and validated a constructed “medicine–compound–target–disease” biological network and enhanced the accuracy of prediction models. Molecular interaction technology, as one of the important emerging technologies, includes biofilm interference technology, plasma resonance technology, and nanoliquid chromatography–mass spectrometry analysis technology (Hsieh and Korfmacher, 2006; Wartchow, et al., 2011; Guo, 2012). High-throughput technology allows for automatic operation and precise processing of cell experiments, quickly obtaining much useful information. Some studies confirmed that network pharmacology was more effective in revealing the principle of small-molecule regulation by using high-throughput technology when constructing a “complex protein–disease” network (Zhang, et al., 2019a). Omics technology, including genomics, proteomics, and metabolomics, more intuitively observes the different regulatory effects of single or compound Chinese medicines at different levels. As an emerging scientific technique, proteomics effectively reveals potential target proteins or protein biomarkers, transforming the promises of TCM into powerful modern therapies (Suo, et al., 2016). These emerging technologies play a strong supporting role in the construction of a biological network, transforming the complex connections and information within the network into a more intuitive visual network, reducing the difficulty of text information, and allowing for further study and research.

#### Network pharmacology research steps and the construction of a biological network

**FIGURE 2:**
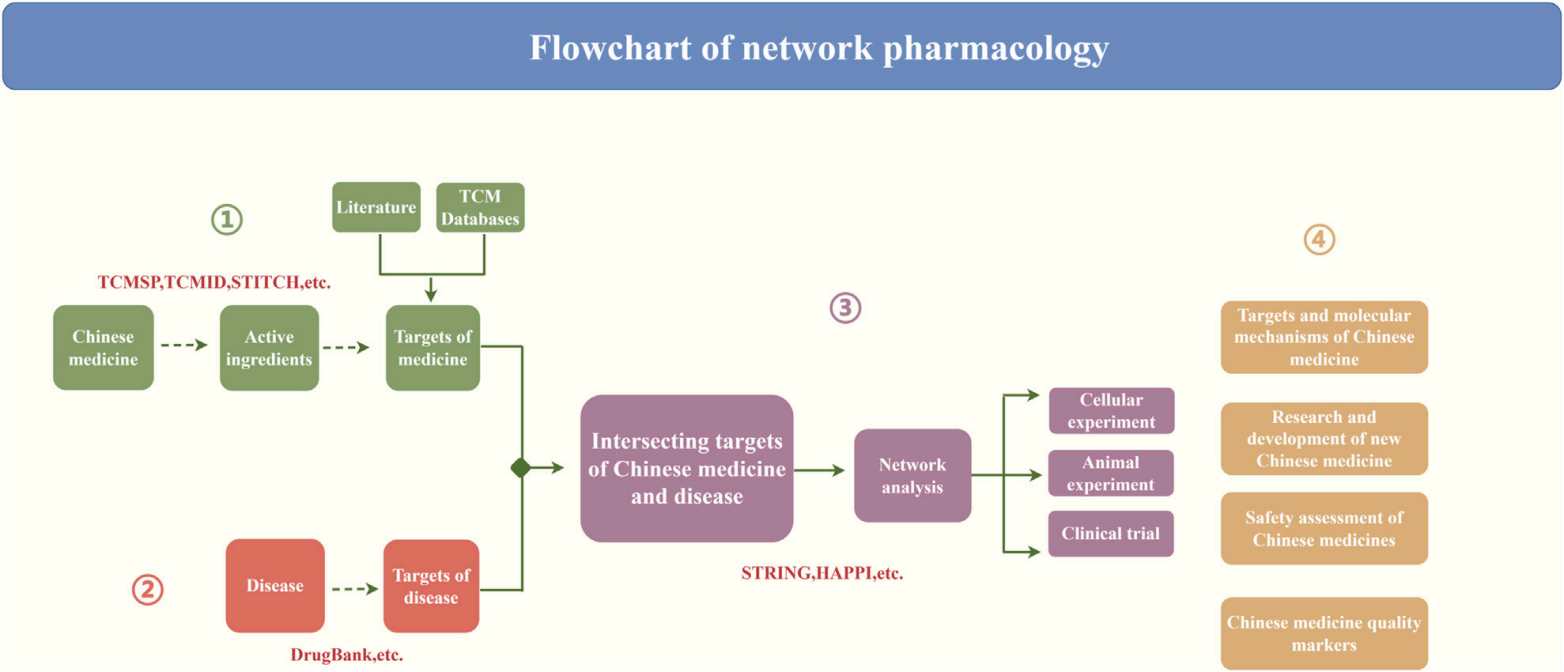
Flowchart of network pharmacology analysis.

The key problem of pharmacologic analysis of the TCM network was evaluating the synergistic effect of a single medicine or multiple target genes of TCM compounds on disease-related molecular networks (Cao, et al., 2023). Current TCM network pharmacology research methods are divided into several steps. First, the key active ingredients of TCM are screened, followed by further screening of the active ingredient of the target gene. Second, the disease-related targets are screened out. Finally, the “medicine–compound–target–disease” network is constructed, and the intersecting targets are subjected to network analysis and relevant experimental validation. Ultimately, it will be applied to the research and development of new Chinese medicines, the safety assessment of Chinese medicines, and so on (Figure 2). In addition to constructing the “medicine–compound–target–disease” network, the interactions between medicines and targets and those between the targets themselves are also crucial. Constructing these relationships further enriches the connotation and significance of biological networks.

#### Discovery of new indications

Through the construction of a biological network, network pharmacology can obtain the compounds, targets, and related signaling pathways of TCM and explore and speculate on new indications for possible TCM treatment based on “unfamiliar” signaling pathways. The characteristics of TCM in selecting appropriate treatment methods according to pathogenesis are consistent with the idea of precision treatment in modern medicine. This provides a reference for the exploration of new treatment methods and new medicine research and development, in line with the development direction of modern medicine.

#### Limitations of network pharmacology

Network pharmacology has emerged as a promising approach to the study of TCM, offering a novel perspective on the multi-compound and multi-target nature of TCM drugs. However, its application has significant limitations. First, network pharmacology and molecular docking experiments, as bioinformatics methods, cannot directly reflect the dose–effect relationship, which hinders the accurate evaluation of therapeutic effects (Li, et al., 2022). Second, the reliance on large datasets means that underreported critical targets may leave essential pathways undetected, and key targets and pathways often lack experimental validation. Third, TCM-specific databases, while relevant, are limited by redundancy and incomplete data, further complicated by the alias problem of medicinal herbs. Despite these challenges, broader databases should be considered in future studies. Fourth, network pharmacology predictions require further experimental validation to confirm the mechanisms of TCM in diseases. Fifth, the incompleteness of data on medicines, genes, and proteins, coupled with insufficient computational software, restricts network pharmacology from making reasonable predictions that need rigorous verification. Finally, determining whether herb–target correlations are direct or indirect and whether the interactions are positive or negative remains difficult, necessitating further experimental investigation. Thus, while network pharmacology offers valuable insights, integrating extensive data resources and rigorous experimental validation is essential to enhancing the reliability and depth of TCM research.

## Application of network pharmacology in digestive diseases

### Application of Chinese medicine in the treatment of chronic gastritis

Chronic gastritis, which is divided into chronic atrophic gastritis and chronic non-atrophic gastritis, is characterized by inflammatory changes in the gastric mucosa due to one or more risk factors. Common symptoms include mucosa atrophy and a rough appearance under the gastroscope. The incidence and detection rates increase with age and are common in clinical settings. It is conservatively estimated that more than half of the world’s population suffers from this disease to some degree (Sipponen and Maaroos, 2015). Chronic atrophic gastritis and chronic non-atrophic gastritis are two different forms and phenotypes of chronic gastritis at different stages of the same disease (Schindler, 1966; Correa, 1988; Price, 1991; Siurala, 1991; Dixon, et al., 1996). There is evidence that the eradication of *Helicobacter pylori* can restore normal gastric mucosa and play a significant role in the cure of chronic gastritis (Valle, et al., 1991; Hojo, et al., 2002; Arkkila, et al., 2006). Chronic gastritis, the most common disease of the digestive system, is often treated with Chinese medicines, with good results in clinical practice.

Yang et al. identified that *Atractylodes macrocephala Koidz* (AMK), a Chinese medicine, had a significant effect on treating chronic gastritis. Using network pharmacology analysis, 27 effective compounds were selected for the treatment of chronic gastritis, forming a network of “medicine–compound–gene–disease”. They identified 26 related signaling pathways, and the reliability of these active ingredients and targets was finally confirmed through *in vitro* experiments. AMK plays a role in the treatment of chronic gastritis by affecting energy metabolism, inflammatory response, and amino acid synthesis (Yang, et al., 2020). Zhou found that network pharmacology, such as the proprietary Chinese medicine Moluodan (MLD) treatment of chronic atrophic gastritis in cell differentiation, apoptosis, cell proliferation, etc., and with the body’s immune response, is associated with inflammation, and it can effectively suppress the GES–1 cell proliferation induced by MNNG, further promote cell differentiation and apoptosis, eventually reduce the inflammation of the stomach, and relieve chronic gastritis symptoms (Zhou, et al., 2022). The clinical empirical formula of Huazhuojiedu decoction, used to treat chronic atrophic gastritis, had 20 key targets that play crucial roles in the treatment, which are closely related to tumor-related pathways and cell proliferation. Animal experiments confirmed that the level of AKT1 increased in model rats. The Huazhuojiedu formulas significantly inhibited AKT1, confirming their therapeutic effect in chronic atrophic gastritis and preventing gastric cancer (Hao et al., 2020). Zhou et al. confirmed that the commonly used formula Lianpu Drink, primarily composed of *Coptis chinensis* and *Magnolia officinalis*, alleviated chronic gastritis symptoms by constructing the “compound–target–disease” network and screening common targets. They confirmed through animal experiments that Lianpu Drink played its role through the RIP/P38 pathway and inhibition of MKK6/P38 (Zhou, et al., 2021a).

To reduce the incidence of gastric cancer developed from chronic gastritis, Wang et al. conducted a network pharmacology study on the traditional Sijunzi formula and found that it mainly treated chronic gastritis by acting on 18 target genes. GO analysis showed that these targets are mainly enriched in the reactions of cells to medicines, the nitric oxide biosynthesis process, the MAPK cascade, angiogenesis, etc. Finally, it is concluded that the Sijunzi formulae reduce the incidence of gastric cancer by improving the inflammation of peripheral blood white blood cells and local inflammation of the stomach (Wang, et al., 2020). Based on the network pharmacology research method for blood absorption ingredients, Li et al. (2018) found that the main ingredients of the Fufang-Xialian capsule, berberine, wogonin, and glycyrrhizin, played an important role in the treatment of chronic atrophic gastritis, and this new method is more straightforward than the traditional network pharmacology approach. These studies confirmed that TCM played an important role in the treatment of chronic gastritis. The research and development of medicines for the treatment of chronic gastritis could fully utilize the network pharmacology research platform, combining the characteristics and advantages of TCM with the latest advancements in science and technology. The specific mechanisms and material basis of TCM and its compound prescriptions in treating chronic gastritis have turned the disadvantages of complex chemical compounds and unclear pharmacological mechanisms of TCM compounds into advantages.

### Application of Chinese medicine in the treatment of ulcerative colitis

Ulcerative colitis (UC), another common digestive system disease, often has a poor prognosis. UC is an inflammatory bowel disease characterized by abdominal pain, diarrhea, mucus pus, and bloody stool as the main clinical manifestations. Studies confirmed that there is no significant difference in the incidence of ulcerative colitis in men and women, but it mainly occurs in young or middle-aged individuals (Cosnes, et al., 2011; Molodecky, et al., 2012). Modern medicine believes that the etiology and pathological mechanisms of UC include environmental factors, impaired immune response, epithelial barrier defects, or microbial interference (Ungaro, et al., 2017; Ramos and Papadakis, 2019). The prevalence and incidence of UC are on the rise, the treatment is more difficult, and the age at onset is less, causing significant inconvenience to patients’ work and lives (Ng, et al., 2017). Commonly used medicines for the treatment of UC in modern medicine mainly include immunosuppressants and aminosalicylic acids such as mesalazine, but their long-term use will cause adverse side effects and damage to the patient’s body (Wan, et al., 2014). Recently, Chinese medicine has played a significant role in treating UC. Academics have increasingly researched the treatment of such inflammatory bowel diseases from the perspective of TCM, achieving greater gains.

The classic prescription Zuojin pill has been known to have the potential to prevent UC since ancient times, but its effective mechanism of action is unclear. The network pharmacology analysis of the Zuojin pill found that the 26 key targets for treating the disease were closely related to the inflammatory response, apoptotic process, signaling, and immune response of UC. By acting on the MAPK signaling pathways, PI3KAkt signaling pathway, prolactin signaling pathways, and toll-like receptor signaling pathways involved in the treatment of UC, it effectively plays an anti-inflammatory role, maintains the integrity of the intestinal mucosa epithelial barrier, and better regulates the intestinal flora. The study reflects the multiple compounds, multiple targets, multiple pathways, and the characteristics of comprehensive control, and it will better guide clinical practice in the future (Wei, et al., 2021). Turmeric, widely used in treating UC, colon cancer, malaria, and Crohn’s disease, was found to have 54 overlapping targets in the treatment of UC. These targets played a therapeutic effect by acting on the MAPK signaling pathway, PI3K-Akt signaling pathway, and JAK-STAT signaling pathway, which are highly correlated with inflammatory bowel disease. Experiments confirmed that *turmeric* could effectively relieve UC pathological manifestations, reduce the expression of STAT3 and TNF-α, and relieve intestinal inflammation by inactivating epithelial cell signal transduction in the *Helicobacter pylori* infection pathway, TNF pathway, and inflammatory bowel disease pathway (Liu, et al., 2021b).

In China, Huiyangjiuji formulas (HYJJD) were widely used in the treatment of inflammation, including UC, but their potential therapeutic mechanism was not completely clear. Some scholars identified the active ingredients of HYJJD through network pharmacology analysis. KEGG pathway analysis revealed that the target genes were enriched in the inflammatory pathway, pathogen-induced infection pathway, and tumor-related pathway. *In vitro* experiments also confirmed that HYJJD had significant inhibitory effects on interleukins such as IL-10, IL-2, and IL-12. Moreover, it promoted intestinal mucosal healing, effectively repaired the damaged IO function of TNF-α, re-established intestinal epithelial homeostasis, and further played an anti-inflammatory role (Yu, et al., 2021). *Paeonia lactiflora*, a common Chinese medicine used to treat dysentery, was found through network pharmacology studies to have 70 common target genes with UC, involving biological functions such as reaction to medicines, reaction to lipopolysaccharides, positive regulation of nitric oxide biosynthesis, and reaction to estradiol. By acting on the TNF signaling pathway, cancer signaling pathway, hepatitis B, tuberculosis, and other signaling pathways, it played a role in treating inflammatory bowel disease and relieving UC symptoms (Zhang, et al., 2019b). Experiments by Zhang et al. (2021b) found that Schisandrin B, the main ingredient of *Schisandra chinensis*, could not only relieve intestinal inflammation but also suppress the leucine-rich repeat, pyrin domain-containing 3 (NLRP3) inflammasome, and nucleotide-binding oligomerization domain in *in vivo* and in vitro models of colitis. A new study found that there are 50 crossover genes between nine compounds in Xianglian pill (XLP) and UC, which mainly reduced mucosal inflammation and exerted anti-inflammatory effects by regulating the relevant pathways of the immune system (Liu, et al., 2021a). The study by Chen et al. on Huangtu decoction (HTD) found that HTD could significantly improve the pathological damage in the colon in UC mice, reduce the serum levels of IL-6 and IL-1β in mice, and finally relieve their symptoms (Chen, et al., 2021). Studies have shown that there are 26 active compounds and 148 key targets in Guchang Zhixie Wan (GZW), which are closely related to UC, and GZW was able to exert a therapeutic effect on UC by acting on inflammation, immune and oxidative stress-related pathways (Zhang, et al., 2021a). According to the above network pharmacology study on single or compound Chinese medicines, the mechanism of TCM in preventing and treating UC might be achieved by regulating the expression of inflammatory factors, changing the quantity and abundance of intestinal microbiota, and regulating the intestinal immune balance. The clinical efficacy was clear, but there are problems with complex ingredients and synergistic effects. Currently, its mechanism of action is not completely clear, and further exploration is still required.

### Application of Chinese medicine in the treatment of gastric cancer

As the most common disease of the digestive system, chronic gastritis could progress to intestinal metaplasia or intraepithelial neoplasia in the epithelium and the glands of the gastric mucosa under the action of *H. pylori* infection, adverse environmental conditions, and an unhealthy diet and eventually develop into gastric cancer. Gastric cancer refers to a malignant tumor arising in the gastric mucus membrane, and clinical symptoms include epigastric ache, emaciation, anorexia, anemia, and an apparent bump that can be touched in the epigastric region when terminal. *Helicobacter pylori* infection and the inclusion of preserved foods in the diet are the main causes of gastric cancer (Johnston and Beckman, 2019). Gastric cancer ranks first among digestive system cancers and third in cancer fatality rates. It predominantly affects the elderly, and the ratio of its incidence in men and women is approximately 2:1. In recent years, the diagnosis rate of gastric cancer in East Asia and other regions with inadequate medical equipment accounts for approximately half of the global diagnosis rate of gastric cancer, while the incidence in Western countries is relatively low. Therefore, regular screening plays a crucial role in gastric cancer prevention. Modern medicine generally treats gastric cancer with surgery, and some doctors use endoscopic treatment, which has the advantages of fewer adverse reactions and good effects. Additionally, chemotherapy could alleviate the symptoms of gastric cancer. TCM also has unique advantages in the treatment of gastric cancer, significantly improving the patient’s quality of life, prolonging survival, and causing fewer side effects. The application of network pharmacology to analyze the mechanism of TCM in the prevention or treatment of gastric cancer could provide new ideas for the future prevention and alleviation of gastric cancer symptoms.

Qu et al. found through TCMSP and other databases that the ZhiShi–BaiZhu herb pair mainly treated gastric cancer through 27 key active compounds and 120 targets, which exerted therapeutic effects on the disease by acting on the IL17 signaling pathway and the PI3K-Akt signaling pathway; the main targets include MMP9, BCL2, MDM2, AKT1, and MOTR; and the final molecular docking showed that the two key ingredients of naringin and luteolin could stably bind to the target (Qu, et al., 2021). Studies have found that the main compounds of *Citri reticulatae pericarpium pinelliae rhizoma* (CRP-PR) include naringin and stigmasterol. The main targets of CRP-PR obtained from the STRING database are JUN, GSK3B, SMAD2, GAPDH, and STAT3, involving 118 cellular components, 540 biological processes, and 171 molecular functions. It was predicted that CRP-PR could exert a therapeutic effect through the MAPK signaling pathway, the PI3K-Akt signaling pathway, and the TNF signaling pathway and exhibited a multi-compound, multi-target, and multi-pathway therapeutic effect of TCM in treating diseases by using the network pharmacology analysis method (Song, et al., 2021). In the study of *Hedyotis diffusa Willd* (HDW), Liu et al. screened 32 active ingredients and 353 key targets. Through network analysis, AKT1, MAPK1, CDK2, PIK3CA, and VEGFA were found to be the key targets of HDW in the treatment of gastric cancer. GO enrichment analysis and KEGG pathway enrichment analysis showed that HDW might act on the VEGF signaling pathway and PI3K/AKT/mTOR signaling pathway through synergistic regulation of cell apoptosis, angiogenesis, and cell differentiation and proliferation, thereby playing a therapeutic role in gastric cancer (Liu, et al., 2018). As a TCM prescription for chronic gastritis, Zuojin pill also had a therapeutic effect on gastric cancer. Network pharmacological analysis showed that Zuojin pill contained 47 key compounds, including quercetin, isorhamnetin, and β-sitosterol, and 48 targets related to gastric cancer. Protein–protein interaction analysis revealed that MMP1, MMP9, and MMP3 played key roles in the anti-gastric cancer mechanism, furthering our understanding of the mechanism of TCM compounds in treating gastric cancer (Zhang, et al., 2020).

Taraxasterol (TAX) (Chen, et al., 2020), with strong antitumor activity, was found to have five common targets with gastric cancer, namely, EGFR, MMP2, AKT1, BRAF, and FGFR2, through network pharmacology methods and animal experiments. TAX prevented further deterioration of gastric cancer by inhibiting the EGFR/AKT1 signaling pathway. Han and Han (2021) found that there are six medicinal compounds and 29 target genes related to gastric cancer in Herba Sarcandrae, which ultimately inhibit further development of gastric cancer by acting on the IL-17 signaling pathway, NF-κB signaling pathway, and related cancer pathways. This provides a new basis for future research on Herba Sarcandrae’s anti-tumor properties. Huang et al. (2020) found that gentiopicroside also plays an important role in the treatment of gastric cancer, and its anticancer activity may involve 53 related targets, confirming that key compounds affect the expression of CCNE1, p-P38, CCND1, and p-AKT at the protein level. The above studies have proven that the mechanism of action of TCM in treating gastric cancer or alleviating adverse symptoms has certain scientific significance, and the application of network pharmacology can better demonstrate the multi-compound, multi-target, and multi-pathway characteristics of TCM in treating diseases, providing a guarantee for future research and development of anti-cancer medicines.

### Application of Chinese medicine in the treatment of liver cirrhosis

As an essential functional organ of the digestive system, the liver undertakes important functions such as metabolism and detoxification. Under the influence of various pathogenic factors, the liver experiences chronic inflammation, steatosis, hepatocyte reduction, and diffuse fibrosis, which can gradually develop into cirrhosis. Cirrhosis is a chronic liver disease characterized by diffuse fibrosis, regenerated nodules, and pseudolobule formation. The main clinical manifestations are portal hypertension and liver dysfunction, with many complications and a poor prognosis. Previous studies found that cirrhosis is the ultimate common pathologic pathway of liver damage caused by multiple chronic liver diseases (Melato and Mucli, 1989; Qua and Goh, 2011; Asrani, et al., 2013). In Western countries, common causes of cirrhosis include viral hepatitis infection, alcoholism, and non-alcoholic fatty liver disease, and improper care can easily transform cirrhosis into liver cancer (Di Bisceglie, 2000; Innes, et al., 2013). Modern treatment focuses on addressing the underlying causes to prevent further liver damage, using anti-liver fibrosis and hepatocyte protection medicines, and actively managing complications such as ascites. Given the serious complications and high mortality associated with cirrhosis, treating complications such as gastrointestinal bleeding is crucial. Network pharmacology analysis shows that TCM treatment of liver cirrhosis also has significant effects and can effectively inhibit its progression to liver cancer.

*Citri Reticulatae Pericarpium* (CRP), a Chinese medicine commonly used for treating liver diseases, contains a variety of flavonoids, including cinnamon and hesperidin. Previous studies found that CRP had anti-tumor and antibacterial effects, reduced cholesterol, and prevented fatty liver disease. Network pharmacology studies revealed that CRP played a significant role in preventing liver injury in 117 targets; GO enrichment analysis includes 139 molecular functions, 1,525 biological processes, and 55 cellular components and KEGG pathway enrichment analysis screened out 49 related pathways, including the PI3K-Akt signaling pathway and IL-17 signaling pathway. CRP alleviates liver injury by affecting inflammation, apoptosis, and energy metabolism, effectively reducing liver fibrosis and preventing cirrhosis (Wu, et al., 2021b). Zhou et al. constructed a “TCM–compound–target” network for Xiaoyaosan decoction (XYS) in treating liver fibrosis, including 8 TCM drugs, 108 active compounds, and 42 key targets. Enrichment analysis showed that AMPK, FoxO, PPAR, TGFβ, MAPK, and other key targets, as well as the hepatitis B and C pathways and the Akt/FoxO signaling pathway, participated in the anti-fibrotic effects of XYS, effectively preventing cirrhosis (Zhou, et al., 2021b).

In the analysis of Yinchenhao decoction (YCHD), Cai et al. (2019) found that it has 45 active compounds and 296 potential targets, which showed an anti-liver fibrosis effect by regulating the PI3K-Akt signaling pathway and the TNF signaling pathway, and animal experiments confirmed that YCHD can effectively reduce the apoptosis of liver parenchymal cells and alleviate the symptoms of liver fibrosis. Network pharmacology studies by Wu et al. confirmed that the Angelicae *Sinensis Radix* (Danggui) and *Ligusticum* Chuanxiong Rhizoma (Chuan Xiong) (DC) herb-pair was commonly used for pain relief and vascular disease prevention, and it had 13 key compounds and 46 anti-fibrosis targets. By acting on the MAPK signaling pathway, it can significantly reduce inflammatory cell infiltration, bile acid level, and collagen deposition caused by CCl4, significantly reduce liver inflammation, and effectively alleviate symptoms of liver fibrosis (Wu, et al., 2021a).

As a common Chinese herbal medicine, *Fructus schisandrae* has been found to have significant hepato-protective effects in clinical applications. A previous study showed that *schisantherin, schisandrin B, schisandrol B, kadsurin*, etc., had significant effects on treating liver disease, especially fatty liver disease, while PPARα, CYP2E1, and AMPK genes and related pathways might play a very important role in the hepatoprotective effects of *F. schisandrae* (Hong, et al., 2017). *Astragalus flavonoids*, present in *Astragali Radix* (AR), have a significant inhibitory effect on liver fibrosis (An, et al., 2021). An et al. found that AR flavonoids could inhibit transforming growth factor-beta 1 (TGF-β1)-mediated activation of hepatic stellate cells (HSCs) and reduce extracellular matrix deposition in HSC-T6 cells by regulating the NF-κB signaling pathway (An, et al., 2021). In addition to the above mechanisms, there are various factors and signaling pathways that are being studied and have not yet been discovered (Chen, et al., 2013). Faced with the complex situation of the current treatment strategies for liver cirrhosis, it is necessary to effectively use TCM to identify syndromes to treat the disease. Combining the advantages of TCM in treating liver cirrhosis with network pharmacology technology provides new solutions and ideas for future treatment approaches (Table 2).

**TABLE 2:** Network pharmacology analysis of TCM in the treatment of digestive system diseases.

References	Disease	Formulas and herbs	Objects	Main compounds	Main target	Main signaling pathway	Outcome
Yang et al. (2020)	Chronic gastritis	AMK	RAW 264.7 cells	Glycine, Arginine L-tyrosine	PTGS1 PTGS2 CHRM3	IL-17 TNF AGE-RAGE	*In vitro* experimental studies have shown that AMK is likely to influence energy metabolism, amino acid synthesis, and inflammatory response when treating CG.
Zhou et al. (2022)	Chronic gastritis	MLD	Cells	Alisol F Scoparone Scopoletin	CDK4 CPT1A NFKB1	T-cell receptor B cell receptor Toll-like receptor	MLD can promote cell apoptosis and differentiation, inhibit cell proliferation, promote lipid droplet accumulation in MNNG-induced GES-1 cells, and reduce the inflammation level.
Hao et al. (2020)	Chronic gastritis	HZJD	SD rats	2,6,2′,4′-tet rahydroxy-6′-methoxychalcone, Acacetin Rivularin	MAPK1, AKT1, and TNF	TNF MAPK P13-Akt	HZJD exerts a therapeutic effect by inhibiting AKT1 in CAG and effectively prevents the occurrence of gastric cancer.
Zhou et al. (2021a)	Chronic gastritis	LPD	SD rats	Quercetin Berberine	IKBKG TRAF6 CTNNB1	TNF MAPK Toll-like receptor	It is through the RIP/P38 pathway and inhibition of MKK6/P38 that LPD plays its role in alleviating the symptoms of chronic gastritis.
Wang et al. (2020)	Chronic gastritis	Sijunzi Decoction	None	Hederagenin 3β-acetoxyatractylone	EGFR TP53 COX2	HIF-1 TNF	Sijunzi decoction alleviates chronic gastritis by inhibiting inflammation and oxidative stress, and it can reduce the incidence of gastric cancer.
Li et al. (2018)	Chronic gastritis	FXL	SD rats	Berberine Wogonin Glycyrrhizin	EGFR TNF IL1B	MAPK TNF VEGF	The main compounds of FXL, berberine, wogonin, and glycyrrhizin play an important role in the treatment of CAG.
Wei et al. (2021)	Ulcerative colitis	Zuojin pill	None	Quercetin Obacunone Berberine	MAPK1 TNF PIK3CA	MAPK Toll-like receptor PI3K-Akt	Zuojin pill can regulate the intestinal flora, maintain the integrity of the intestinal mucosal epithelial barrier, and exert anti-inflammatory effects.
Liu et al. (2021a)	Ulcerative colitis	*Curcuma*	Molecular docking	CLR Campesterol Stigmasterol	TNF-α AKT1 STAT3	JAK-STAT MAPK PI3K-Akt	*Curcuma* can alleviate the pathological manifestations of UC and reduce the expression of STAT3 and TNF-α.
Yu et al. (2021)	Ulcerative colitis	HYJJ	C57BL/6 mice	Hederagenin Karanjin Beta-sitosterol	IL-10 IL-2 IL-12	TNF Nod-like receptor Toll-like receptor	HYJJ re-establishes homeostasis of the gut epithelium during colitis by orchestrating cytokine interaction and suppressing inflammation.
Zhang et al. (2019b)	Ulcerative colitis	*Paeonia lactiflora*	None	Kaempferol Beta-sitosterol (+)-Catechin	IL6 BCL2 AKT1	Pathways in cancer TNF Tuberculosis	By acting on the TNF signaling pathway, cancer signaling pathway, hepatitis B, and other signaling pathways to play a role in the treatment of inflammatory bowel disease.
Zhang et al. (2021a)	Ulcerative colitis	*Schisandra chinensis*	C57BL/6 mice	Schisandrin B	TNF-α IL-1β IL-6	AMPK/Nrf2	Schisandrin B can not only relieve intestinal inflammation, but also suppress the leucine-rich repeat, pyrin domain-containing 3 (NLRP3) inflammasome, and nucleotide-binding oligomerization domain *in vivo* and vitro model of colitis.
Liu et al. (2021b)	Ulcerative colitis	XLP	SD rats	Jatrorrhizine Berberine Coptisine	JAk2 STAT3 HIF-1α	Th17 cell differentiation JAK-STAT PI3K-Akt	XLP can reduce mucosal inflammation and exert anti-inflammatory effects by regulating the relevant pathways of the immune system.
Chen et al. (2021)	Ulcerative colitis	HTD	Female Swiss mice	Glycyrrhizic acid Wogonin Liquiritigenin	IL-6 IL-1β MMP1	Pathways in cancer IL-17	HTD can improve the pathological damage of the colon in UC mice and reduce the serum levels of IL-6 and IL-1β in the serum of mice.
Zhang et al. (2021a)	Ulcerative colitis	GZW	SD rats Normal mouse RAW 264. 7 cells	Beta-sitosterol Kaempferol Quercetin	IL-1α IL-1β STAT1	STAT3 NF-kB IL-6	GZW’s targets play a role in UC through inflammatory-, immune-, and oxidative stress-related pathways.
Qu et al. (2021)	Gastric cancer	ZS-BZ herb pair	Molecular docking	Naringenin Luteolin	MMP9 BCL2 MDM2	IL17 PI3K-Akt	ZS–BZ herb pair can play a therapeutic role in treating gastric cancer based on the active compounds naringenin and luteolin.
Song et al. (2021)	Gastric cancer	CRP-PR	TCGA and HPA database	-)-Alpha-pinene ()-Cuparene EIC	GAPDH MAPK3 JUN	PI3K-Akt MAPK	CRP-PR can exert its therapeutic effect on UC through the MAPK signaling pathway, PI3K-Akt signaling pathway, and TNF signaling pathway.
Liu et al. (2018)	Gastric cancer	HDW	None	Quercetin Coumarin	AKT1 MAPK1 CDK2	VEGF PI3K/AKT/mTOR	HDW can act on the VEGF signaling pathway and PI3K/AKT/mTOR signaling pathway through synergistic regulation of cell apoptosis, participation in angiogenesis and cell differentiation and proliferation, and, thus, further play a therapeutic role in gastric cancer.
Zhang et al. (2020)	Gastric cancer	Zuojin pill	Molecular docking	Quercetin Isorhamnetin β-sitosterol	MMP1 MMP3 MMP9	IL-17 Toll-like receptor	The key targets of Zuojin pill MMP1, MMP3, and MMP9 play a therapeutic role in gastric cancer.
Chen et al. (2020)	Gastric cancer	Dandelion	6-week-old nude mice	Taraxasterol	EGFR MMP2 AKT1	EGFR/AKT1	TAX prevents further progression of gastric cancer by inhibiting the EGFR/AKT1 signaling pathway.
Han and Han. (2021)	Gastric cancer	Herbal *Sarcandrae*	TCGA database	Beta-sitosterol Sitosterol Engeletin	IL-6 MMP9 HMOX1	IL-17 NF-κB	Herba *Sarcandrae* inhibits the further development of gastric cancer by acting on the IL-17 signaling pathway, NF-κB signaling pathway, and related cancer pathways.
Huang et al. (2020)	Gastric cancer	*Gentain*	Cell lines	Gentiopicroside	CCND1 AKT CCNE1	Metabolic PI3K-Akt	Gentiopicroside affects the expression of CCNE1, p-P38, CCND1, and p-AKT at the protein level, thereby exerting its anti-gastric cancer effect.
Wu et al. (2021b)	Liver cirrhosis	CRP	LX-2 cell line	Hesperidin Naringenin	CASP3 BAX BCL2	PI3K-Akt IL-17	CRP alleviates liver injury by affecting inflammation, apoptosis, and energy metabolism, and it can effectively alleviate liver fibrosis symptoms and prevent the occurrence of liver cirrhosis.
Zhou et al. (2021b)	Liver cirrhosis	XYS	Wistar rats	Beta-sitosterol Stigmasterol Hederagenin	AMPK FoxO PPAR	Hepatitis B Hepatitis C Akt/FoxO	In histopathological and serum liver function analyses, XYS markedly alleviated symptoms of CCl4-induced liver fibrosis.
Cai et al. (2019)	Liver cirrhosis	YCHD	Wistar rats	Kaempferol Areapillin Quercetin	BAX CASP8 CASP3	PI3K-Akt TNF	YCHD can effectively reduce the apoptosis of liver parenchymal cells and alleviate the symptoms of liver fibrosis.
Wu et al. (2021b)	Liver cirrhosis	DC herb-pair	C57BL/6 mice	Ferulic acid β-sitosterol Stigmasterol	AKT EGFR STAT3	MAPK	DC herb-pair can significantly reduce inflammatory cell infiltration, bile acid level, and collagen deposition caused by CCl4, significantly reduce liver inflammation, and effectively improve liver fibrosis.
Hong et al. (2017)	Liver cirrhosis	*Fructus Schisandrae*	Molecular docking	Schisantherin Schisandrin B Schisandrol B	PPARα CYP2E1 AMPK	MAPK AMPK	PPARα, CYP2E1, and AMPK genes and their related pathways may play a very important role in the hepatoprotective effects of *Fructus schisandrae.*
An et al. (2021)	Liver cirrhosis	*Astragali Radix*	HSC-T6	Calycosin Formononetin Kaempferol	AKT1 TP53 MAPK3	NF-κB IL-17 TNF	The flavonoids from AR were able to suppress TGF-β1-mediated activation of HSCs and reduce extracellular matrix deposition in HSC-T6 cells via regulation of the NF-κB signaling pathway.

## Conclusion and future perspectives

In conclusion, TCM has played an irreplaceable role in treating digestive system diseases, and it is closely related to the holistic concepts of TCM. Despite its significant advantages, TCM faces limitations and challenges in clinical practice. The individualized nature of TCM treatments results in poor reproducibility, with the complex composition and lack of standardization of herbal medicines hindering widespread adoption. The theoretical differences between TCM and modern medicine further complicate research efforts. Future progress depends on the modernization and internationalization of TCM, which requires enhanced basic research, modern technological applications to elucidate mechanisms of action, large-scale clinical trials to confirm efficacy and safety, and robust quality standards for herbal medicines. Additionally, the application of TCM in treating digestive diseases is controversial, with skepticism from some in the Western medical community due to the perceived lack of scientific evidence, and cultural and philosophical differences contributing to the debate. Addressing these concerns through scientific methods and data is essential for fostering integration between TCM and Western medicine, ultimately improving patient care.

Network pharmacology technology has significant advantages in the field of TCM research, such as discovering the basis of pharmacodynamic substances, developing new indications, studying the mechanism of medical action, and studying the compatibility of TCM. The information network provides a new research direction for basic research on digestive system diseases. Under the guidance of the network pharmacology evaluation method guidance, the development of network pharmacology is expected to become increasingly healthy and standardized, and the network targets will help produce more high-quality research results for TCM and effectively promote the internationalization and modernization of TCM (Wang, et al., 2022b). However, the results of network pharmacology research are still affected by analysis methods, screening conditions, and database information. The system uses the common intersection of targets between medicines, and there are limitations in research aimed at using medicine synergy. In addition, network pharmacology ignores the influence of the amount of each compounds in TCM, and it is urgent to design a corresponding network algorithm to quantitatively evaluate how medicines work. These are challenges in the discovery of TCM combinations and new medicines, which require further research.

Importantly, future research must integrate the analysis results with traditional TCM theories to reveal the mechanism of TCM in treating diseases from multiple angles. Combining network data analysis with fundamental medical research can provide more opportunities for the modernization of TCM. In the new era, it is crucial to leverage the precious resource of network pharmacology, systematically combining TCM with the emerging network pharmacology technology. This approach will better explore and utilize TCM’s characteristic theories and rich experience. Integrating bioinformatics technology into specific medical life science research and fostering multidisciplinary cooperation will further advance the research process in diagnosing and treating digestive system diseases.
